# Potential Benefits of Antioxidant Phytochemicals on Endogenous Antioxidants Defences in Chronic Diseases

**DOI:** 10.3390/antiox12040890

**Published:** 2023-04-06

**Authors:** Anna Maria Fratta Pasini, Luciano Cominacini

**Affiliations:** Department of Medicine, Section of Internal Medicine D, University of Verona, 37134 Verona, Italy; luciano.cominacini@univr.it

Chronic diseases and cancer are worldwide health problems which result in death and disability for millions of people [1]. Numerous epidemiological studies and meta-analyses have suggested that many natural phytochemicals display health-promoting advantages, including the prevention of the most common chronic diseases and cancer [2,3,4,5,6,7]. Phytochemicals are a class of compounds designated as bioactive non-nutrient substances contained in fruits, vegetables, grains, and other plants [8]. The protective role of phytochemicals may be associated with their antioxidant and anti-inflammatory activities [9,10], since the overproduction of oxidants (reactive oxygen species and reactive nitrogen species) in the human body is involved in the pathogenesis of many chronic diseases, cancer and aging [11,12,13,14]. In addition, phytochemicals can regulate the expression and transcription of multiple antioxidant-related genes, thereby modulating the activity of antioxidant enzymes and antioxidants, such as glutathione, to counteract oxidative stress [2,12,15,16,17].

It is well known that polyphenols have, in general, poor bioavailability, and it is likely that their biological action is mediated by their metabolites, which have antioxidant and anti-inflammatory resources [18]. Moreover, news is coming that bioavailability is affected by several factors such as food source, food processing, gut microbiome/hepatic biotransformation, and individual variability. For instance, Rodriguez-Mateos et al. [19] showed that the bioavailability of polyphenols was quite different after the consumption of berry drinks compared to a berry bun in healthy volunteers. In addition, very recent data indicate that cooking may also enhance the availability of polyphenols [20]. Heat processing, in fact, produces the release of polyphenols from the food matrix and can augment absorption and colonic concentrations [20]. Furthermore, it has to be underlined that an inter-individual variation in response to the consumption of plant food has been reported [21]. Finally, one therapeutic strategy for improving the favourable effects of polyphenols could be drug synthesis, with the creation of compounds with optimized pharmacokinetic properties. Since there are very important topics that need further deepening, we feel that there is still a need to continue the field of oxidative stress research in chronic diseases and the correlated therapeutic approach, both with diets rich in phytochemicals and antioxidant supplementation. This Special Issue concerning “Potential benefits of antioxidant phytochemicals on endogenous antioxidants’ defences in chronic diseases” contains seven contributions, one review and six research articles.

Hyperglycaemia-induced oxidative stress is central to the development of diabetes and diabetic complications [22]. Notably, diabetic micro- and macrovascular complications significantly worsen the burden of diabetes [23]. In their interesting review, Akpoveso et al. [24] summarized the body of literature on diabetic micro- and macrovascular complications with a focus on (1) the roles of oxidative stress, (2) the activation of endogenous antioxidant defences by exogenous phytochemicals, and (3) the therapeutic potentials of phytochemicals as alternative/adjuvant options. Concerning the implications of oxidative stress, five major metabolic pathways are commonly accepted to contribute to diabetic complications: the formation and accumulation of advanced glycation end-products (AGEs), the increased expression of the receptor for AGEs and its activating ligands, the activation of protein kinase C isoforms, increased flux via the hexosamine pathway, and an increased polyol pathway. These pathways are major key contributors to increased oxidative stress, probably through the overproduction of mitochondrial superoxide by the mitochondrial electron transport chain. In addition, these five pathways, by increasing reactive oxygen species (ROS) generation, cause inflammation, angiogenesis, and vascular anomalies. The ROS increase is not adequately counteracted by the endogenous antioxidant system, which is dysfunctional in hyperglycaemic conditions (a fact that greatly contributes to the development of diabetic micro and macrovascular complications). Finally, the authors reviewed the current literature, showing that polyphenols could reduce oxidative stress and hyperglycaemia, and prevent vascular diabetic complications in human and animal studies. In particular, the authors focused on some endogenous antioxidant systems such as mitochondrial uncoupling protein 2, glutathione peroxidase 4 and coenzyme Q, silent information regulator proteins, and nuclear factor-E2-related factor 2 (Nrf2). The conclusion is that, overall, polyphenols positively influence endogenous antioxidant systems via multiple mechanisms, and therefore become alternative/adjuvant options in the treatment of vascular complications of diabetes. 

Since usual treatments to control obesity-related issues have suggested their non-efficacy and a need for alternative approach, Iftikhar et al. [25] aimed to assess and validate the potential of polyphenol-rich star anise tea (SAT) on oxidative stress, obesity, and related biochemical parameters in a rat high-fat-sugar-diet (HFSD)-induced obesity model. The results obtained by the SAT were compared with those acquired by orlistat, the only medication authorized by the European Medicines Agency for the treatment of chronic obesity. The phenolic profile of SAT showed that the major phenolic acids were p-coumeric acid, gallic aid, cinamic acid, chlorogenic acid and ferulic acid, while catechin and rutin were the major flavonoids detected in the extract. SAT exhibited significant in vitro radical scavenging activity. In the experimental design, the normal control group (NC) received normal feed, the HFSD group received HFSD, the positive control group received HFSD plus orlistat, the SAT-250 group was supplemented with SAT (250 mg/kg BW/day), and the SAT-500 group received HSFD supplemented with SAT (500 mg/kg BW/day). The HFSD group showed a significant increase in body weight and body mass index, validating the study’s adopted model of obesity. Interestingly, both doses of SAT showed an anti-obesity effect that was comparable with orlistat, especially when the higher dose was used. SAT also improved the lipid profile, and the oxidative stress parameters such as malondialdehyde (MDA), superoxide dismutase (SOD), and glutathione worsened by HFSD. The conclusion of this interesting study is that SAT possesses strong protective effects against obesity and oxidative stress, and opens the possibility of using these herbal infusions as beneficial supplementation.

There is evidence showing that metabolic disorders related to obesity and type 2 diabetes are associated with aggravated cerebrovascular damages during stroke [26]. In this context, Taïlé et al. [27] analysed the protective action of the polyphenol-rich extract of *Antirhea (A.) borbonica*, a medicinal plant referenced in the French Pharmacopeia for antidiabetic properties, in a mouse model of stroke exposed to high-fat diet (HFD)-induced obesity and in murine cerebral endothelial cells in high glucose condition. First of all, the authors identified the polyphenols present in the *A. borbonica* plant extract and found that the most abundant polyphenols identified were caffeic acid esters belonging to the family of dietary phenolic acids. Other phenolic acids deriving from coumaric acid and glycosylated derivatives of quercetin and kaempferol were also detected. Mice exposed to HFD exhibited a higher body weight than animals receiving a normal diet and an accumulation of fat deposits. The administration of *A. borbonica* to mice significantly reduced glycemia and attenuated the associated glucose intolerance in the HFD group. Additionally, *A. borbonica* improved dyslipidemia and the systemic anti-inflammatory profile in obese mice. Most importantly, the authors demonstrated that *A. borbonica* counteracted the increase in cerebral infarct volume observed in obese mice. Furthermore, *A. borbonica*, and more particularly caffeic acid, also reduced the haemorrhagic transformation of brain infarct and blood–brain barrier (BBB) disruption. Intriguingly, *A. borbonica* also exhibited antioxidant properties by preserving the activation of the endogenous antioxidant system involving Nrf2 and SOD. In addition, *A. borbonica* extract reduced oxidative stress aggravated by HFD-induced obesity at both the cerebral and peripheral levels during stroke. Finally, the results of this study raise the possibility of MMP-2 involvement in cerebral endothelial cell permeability and BBB disruption during neuroinflammation and oxidative stress aggravated by HFD in obese and hyperglycaemic mice during stroke, and show the protective action of polyphenols in counteracting MMP-2 activity. In conclusion, these findings show that the polyphenol-rich extract of the medicinal plant *A. borbonica* reduced cerebrovascular, inflammatory, and metabolic disorders aggravated by obesity in a mouse model of stroke, and identify new bases to improve the clinical consequences of stroke in the context of obesity and diabetes.

In a further study in this Special Issue, Kim et al. [28], starting from the point that a variety of plant-derived phenolics are known to attenuate cognitive impairment in Alzheimer’s disease by radical scavenging and reinforcing synaptic plasticity activities [29,30], examined the cognition-improving effect of *Pinus densiflora* bark extract (PBE) in Sprague Dawley (SD) rats with scopolamine (SCOP)-induced learning and memory deficits. Since information on *Pinus densiflora* phytochemical profiles was limited, the authors first identified and quantified phenolics in the PBE. Interestingly, a total of 23 phenolic compounds were identified, including hydroxymandelic acid, syringaldehyde, 3-p-coumaroylquinic acid, 4-p-coumaroylquinic acid, quercetin 3-O-rhamnoside (quercitrin), and quercetin 7-O-glucoside (quercimeritrin), which were first identified in this study. Moreover, protocatechuic acid, taxifolin, and procyanidin B dimer and its building block (+)-catechin were the principal components of PBE. Then, the authors assessed whether this phenolic-rich PBE would prevent the deterioration of memory and learning activity in six-week-old male SD rats with SCOP-induced learning and memory deficits. The results show that SCOP-treated SD rats had cognitive deficiency, whereas PBE enhanced cognitive and memory impairment. Interestingly, antioxidant biomarkers and cholinergic function were improved in the hippocampal homogenate of rats fed with PBE. Evidence indicates that the impairment of memory and learning ability due to Alzheimer’s disease is closely related to long term potentiation (LTP) induction failure in the hippocampus [30]. Since oxidative stress status is strongly related to LTP impairment [30], the authors evaluated whether PBE could ameliorate LTP failure using organotypic cultured hippocampal slices from seven-day-old SD rats. Intriguingly, PBE was shown to improve LTP induction and to retrieve LTP from blockades by the muscarinic cholinergic receptor antagonists. In conclusion, this elegant study indicating that PBE has cognition-enhancing effects adds new insights on nutraceutical candidates for the prevention of cognitive disorders.

In the next interesting contribution, Bae et al. [31] tested the hypothesis that phenolic compounds in *Oenanthe (O.) javanica*, a small perennial herb traditionally used for food and various medicinal purposes in East Asian countries, have a positive effect on colitis symptoms and gut microbiota in mice with dextran sulphate sodium (DSS)-induced colitis. First, the authors identified the three main components of *O. javanica*; chlorogenic acid, 5-O-feruloylquinic acid, and quercetin-3-rutinoside. Then, in *in vitro* studies, *O. javanica* ethanol extract (OJE) was shown to inhibit the production of inflammatory cytokines in lipopolysaccharide-stimulated macrophages. Furthermore, OJE supplementation alleviated colitis symptoms in mice with DSS-induced colitis and down-regulated the expression of pro-inflammatory cytokines and the activation of the NF-kB signalling pathway. As a matter of fact, OJE supplementation significantly reduced colonic macrophage infiltration and associated inflammatory markers compared to control mice, indicating that OJE may inhibit the early stages of colonic tissue damage. It has been shown previously that the Nrf2 pathway plays an important role in protecting gut integrity through the regulation of antioxidant enzymes and proinflammatory cytokines produced by oxidative stress in ulcerative colitis [32]. In this study, the protein expression of Nrf2 and of the phase 2 antioxidant enzymes, significantly decreased in DSS-induced colitis, was significantly increased by OJE administration. Since the importance of gut microbiota in the pathogenesis of inflammatory bowel disease is known, the authors finally evaluated the changes in gut microbiota of mice with DSS–induced colitis after OJE supplementation. Interestingly, a taxonomic assignment analysis reported a reduction in the abundance of Proteobacteria, including Escherichia, and an increase in the abundance of the genus Muribaculum, thereby restoring the intestinal microbial communities found in healthy mice. These attractive results, showing that OJE exerts beneficial effects on inflammation and gut microbial composition in a mouse model of colitis, suggest that OJE may become a new phytochemical for the adjuvant treatment of inflammatory bowel disease.

In the following paper, Stranieri et al. [17] evaluated whether polyphenols contained in a peculiar red wine, made from grapes and winemaking techniques designed to achieve a high content of polyphenols, can cross cell membranes and switch the oxidant/antioxidant balance toward an antioxidant pattern by means of a gene regulatory system. First, wine and the cell extracts were analysed with an untargeted metabolomics approach based on ultraperformance liquid chromatography-electrospray ionization-mass spectrometry. The wine metabolites recovered in treated cells belonged, on the whole, to stilbenes and flavan-3-ols derivatives, which corresponded to the more abundant metabolites present in the wine extract. Interestingly, the accumulation of the wine metabolites within the cells was not directly proportional to the abundance of metabolites in the wine extract. For instance, the resveratrol tetramer accumulated much more than the procyanidin P3 type and procyanidin P2 type, which were more abundant in the wine. The authors next assessed whether these intracellular metabolites could counteract ROS generation. The results indicated that wine extract determined a dose-dependent ROS reduction in THP-1 cells and cardiomyocytes. Moreover, the authors evaluated the effect of wine metabolites on the Nrf2/antioxidant response element (ARE) pathway activation. The preincubation of cultured cells with wine extract induced Nrf2 nuclear translocation and counteracted the Nrf2 fall caused by ROS overproduction, both in THP-1 cells and in cardiomyocytes. The rise in Nrf2 was paralleled by the increase in ARE genes hemeoxygenase-1 and glutamate-cysteine ligase’s catalytic subunit expression. In conclusion, the authors demonstrated that particular polyphenol metabolites of red wine can accumulate within the cells in a metabolite-specific manner. This accumulation allows the cells to oppose oxidative stress and upregulate the antioxidant Nrf2/ARE pathway. The results of this elegant study add new insights on the health-related activity of wine polyphenols.

It is well recognized that heavy metal pollution is a significant environmental problem. The use of industrial products that contain cadmium metal is increasing, leading to environmental pollution and increased occupational exposure and toxicity in humans [33]. Exposure to the heavy metal cadmium chloride (CdCl2) in humans can result in negative health effects, such as cardiovascular diseases, alterations in organ function, and hepatotoxicity [33]. CdCl2 is known to cause an imbalance in the oxidant status of the body by triggering the release of ROS [34]. In their contribution, Hamza et al. [35] investigated the effect of *Rosa damascena* (*R. damascena*) extract on oxidative stress, hepatotoxicity, and the injured cardiac tissue of male rats exposed to CdCl2. The analysis of the *R. damascena* extract indicated that the most important components included nonadecane, phthalic acid, gallic acid, and quercetin, i.e., phenolic compounds with strong antioxidant and free radical scavenging activities. Furthermore, the *R. damascena* extract contained apigenin, a natural product belonging to the flavone class and possessing strong antioxidant and anti-inflammatory functions. In their experimental design, the authors used forty male Wistar albino rats divided into four groups: the control group, the CdCl2-treated group, the *R. damascena* extract group, and the combination CdCl2 and *R. damascena* extract group. Rats exposed to CdCl2 showed multiple significant histopathological changes in the liver and heart, including inflammatory cell infiltration and degenerative alterations associated with elevated levels of alanine and aspartate aminotransferase, increased levels of hepatic and cardiac ROS, malondialdehyde (MDA), lipid peroxidation (LPO), tumour necrosis factor-alpha and interleukin-6, and decreased antioxidant defences. Intriguingly, *R. damascena* administration prevented liver and heart injury, suppressed excessive ROS generation, MDA, LPO, and inflammatory responses, and enhanced antioxidant defences. The authors conclude by suggesting that *R. damascena* may be a candidate for attenuating hepatic and cardiac toxicity caused by CdCl2 exposure.

We would like to express our appreciation for the authors who participated in this Special Issue and congratulate them for the outstanding level of their studies. In all the papers, the authors emphasized the role of oxidative stress in the pathophysiology of different chronic diseases and dug deep into the mechanisms underlying the potential benefits produced by phytochemical antioxidants. In addition, there was a general consensus on the fact that further in vivo studies are needed to recommend phytochemical antioxidants as an alternative/adjuvant option in the treatment of chronic diseases.
